# Retraction: Fabrication of Fe_3_O_4_@PVA-Cu nanocomposite and its application for facile and selective oxidation of alcohols

**DOI:** 10.3389/fchem.2026.1802299

**Published:** 2026-02-16

**Authors:** Frontiers Editorial Office

The Journal retracts the 21 July 2020 article cited above.

Following concerns regarding the originality of the article, an investigation was conducted in accordance with Frontiers’ policies. It was found that the complaint was valid and that the article should be retracted because of an unacceptable level of similarity in Figures 1-6 to other articles, most notably:

Maleki, A., Niksefat, M., Rahimi, J., & Taheri-Ledari, R. (2019). Multicomponent synthesis of pyrano [2, 3-d] pyrimidine derivatives via a direct one-pot strategy executed by novel designed copperated Fe3O4@ polyvinyl alcohol magnetic nanoparticles. Materials Today Chemistry, 13, 110-120.

Maleki, A., Rahimi, J., and Valadi, K. (2019). Sulfonated Fe3O4@PVA superparamagnetic nanostructure: design, in-situ preparation, characterization and application in the synthesis of imidazoles as a highly efficient organic–inorganic Bronsted acid catalyst. Nano-Structures & Nano-Objects. 18: 100264. doi: 10.1016/j.nanoso.2019.100264.

This retraction was approved by the Chief Editors of Frontiers in Chemistry and the Chief Executive Editor of Frontiers. The authors do not agree to this retraction.

